# A composite visualization method for electrophysiology-morphous merging of human heart

**DOI:** 10.1186/s12938-017-0368-1

**Published:** 2017-06-08

**Authors:** Fei Yang, Lei Zhang, Weigang Lu, Yue Zhang, Wangmeng Zuo, Kuanquan Wang, Henggui Zhang

**Affiliations:** 1 School of Mechanical, Electrical & Information Engineering, Shandong University, Weihai, China; 2 School of Art and Design, Harbin University, Harbin, China; 3 Department of Educational Technology, Ocean University of China, Qingdao, China; 4 School of Computer Science and Technology, Harbin Institute of Technology, Harbin, China; 5 School of Physics and Astronomy, University of Manchester, Manchester, M139PL UK

**Keywords:** Computational cardiology, Morphous, Biophysical merging, Cardiac biophysiological function, Merging attenuation model

## Abstract

**Background:**

Electrophysiological behavior is of great importance for analyzing the cardiac functional mechanism under cardiac physiological and pathological condition. Due to the complexity of cardiac structure and biophysiological function, visualization of a cardiac electrophysiological model compositively is still a challenge. The lack of either modality of the whole organ structure or cardiac electrophysiological behaviors makes analysis of the intricate mechanisms of cardiac dynamic function a difficult task. This study aims at exploring 3D conduction of stimulus and electrical excitation reactivity on the level of organ with the authentic fine cardiac anatomy structure.

**Methods:**

In this paper, a cardiac electrical excitation propagation model is established based on the human cardiac cross-sectional data to explore detailed cardiac electrical activities. A novel biophysical merging visualization method is then presented for biophysical integration of cardiac anatomy and electrophysiological properties in the form of the merging optical model, which provides the corresponding position, spatial relationship and the whole process in 3D space with the context of anatomical structure for representing the biophysical detailed electrophysiological activity.

**Results:**

The visualization result present the action potential propagation of the left ventricle within the excitation cycle with the authentic fine cardiac organ anatomy. In the visualized images, all vital organs are identified and distinguished without ambiguity. The three dimensional spatial position, relation and the process of cardiac excitation conduction and re-entry propagation in the anatomical structure during the phase of depolarization and repolarization is also shown in the result images, which exhibits the performance of a more detailed biophysical understanding of the electrophysiological kinetics of human heart in vivo.

**Conclusions:**

Results suggest that the proposed merging optical model can merge cardiac electrophysiological activity with the anatomy structure. By specifying the respective opacity for the cardiac anatomy structure and the electrophysiological model in the merging attenuation function, the visualized images can provide an in-depth insight into the biophysical detailed cardiac functioning phenomena and the corresponding electrophysiological behavior mechanism, which is helpful for further speculating cardiac physiological and pathological responses and is fundamental to the cardiac research and clinical diagnoses.

## Background

The cardiac disease, e.g., atrioventricular block, ventricular fibrillation and cardiomyopathy, is one of the most common causes of mortality in the world. It has been proven that cardiac functional abnormity may produce the serious problem of heart disability and are generally life-threatening [1]. Although there are considerable progresses have been achieved on conventional open-heart research and acquirement of experimental and clinical data, the cardiac mechanisms aren’t well understood. Increasing momentum is tending to study the cardiac biophysiological function noninvasively [2–4]. Serpooshan et al. [5] analyzed the structure and function of the failing heart to enhance cardiac healing after injury. Zhang et al. [6] visualized cardiac microvessels embolization in 3D space with X-ray phase contrast images. This method was beneficial to diagnoses and predicting and judging the prognosis in myocardial infarction (MI). Based on the surface shape space Taimouri and Hua [7] et al. defined two novel shape descriptors by the geodesic distance connecting two points in the two cardiac medial surfaces, which quantitatively analyzed the similarity and disparity of the 3D heart motions between the healthy and myopathic subjects and accurately detected myopathic regions on the left ventricle. Spicher et al. [8] proposed a new cardiac triggering method to estimate the cardiac cycle phase in real-time from videos captured with an in-bore camera. However, the function mechanisms and the underlying genesis of human heart still remain unclear.

Electrophysiological behavior is of great importance for analyzing the functional mechanism under cardiac physiological and pathological condition. Noble [9] first applied the cell mathematical model-HH model to the purkinje fibre and pace-maker cells and started the research on cardiac function by modeling of cardiac electrophysiological activities. Zhang et al. [10] proposed mathematical models of action potentials in the periphery and center of the rabbit sinoatrial node. Models of the ventricular action potential [11, 12] were constructed to describe the electrophysiological activity of the single ventricular cell in detail. Based on the experimental data of human heart, Priebe and Beuckelmann [13] established the first human ventricular cell model. ten Tusscher et al. [14] created a new human ventricular cell model which contains all major ion channel currents and thus is more close to the human heart’s real condition in the electrophysiological properties. Hilgemann et al. [15] constructed the first excitation–contraction model in the rabbit atrium. Nygren [16] and Courtemanche proposed [17] common human atrium cell models and the models were improved based on the recent experimental data [18–20]. Salinet et al. [21] presented the autoregressive (AR) spectral estimation techniques to produce 3D dominant frequency (DF) maps of atrial electrograms (AEGs) for persistent atrial fibrillation (persAF) study. Wang and Yang et al. [22, 23] implemented the ventricular ischemic model and visualized the electrophysiological activity. A framework to simulate multi-scale wave propagation of ischemia is proposed [24], which leverages the high-performance computing capacity of Graphic Processing Units (GPU).

Recently, visualization of cardiac models in the organ level have been developed to express the microscopic origin of electrophysiological mechanism in the three dimensional space. Rubio-Guivernau et al. [25] visualized myocardial substrate and located conducting channels for pre-planning and guidance of ablation procedures. Lu et al. [26, 27] developed and visualized the human ventricular ischemic model to analyze the influence of acute global ischemia on cardiac electrical activity and subsequently on reentrant arrhythmic genesis. Detailed structures of the human heart are revealed by visualizing the cardiac volume data [28–33]. Seemann [34] established heterogeneous three-dimensional anatomical and electro-physiological model of human atria. Based on the novel fusion transfer function, Zhang and Wang et al. developed a platform integrating multi-volume visualization method for both heart anatomical data and electrophysiological data visualization [35–37]. However these methods did not demonstrate electrophysiological activity clearly with anatomy. Due to the mass of cardiac tissues and complicated cardiac functional mechanism, visualizing the biophysically detailed cardiac model becomes a more difficult task.

In this paper, firstly a cardiac electrical excitation propagation model is established based on the cardiac cross-sectional data to explore detailed cardiac electrical activities. Then a novel merging attenuation function (MAF) is proposed for the visualization of biophysical merging model of cardiac anatomy structure and electrical excitation reactivity conduction. The description of the cardiac excitation kinetics is thus coupled with a genuine fine anatomical geometry, which provides the corresponding position and 3D spatial relationship in the context of anatomical structure for representing the electrophysiological activity in vivo. By the biophysically detailed visualization image, medical staffs and cardiac researchers can obtain insight into underlying mechanisms by observing regions of particular interest of the biophysical area with real anatomical structure context from an arbitrary perspective, which gives a reliable and optimal pre-computed view for the cardiac research and clinical diagnoses.

The rest of the paper is organized as follows. In “Data and methods”, the human cardiac anatomical data and multicellular tissue action potential conduction model is described. Then the MAF is constructed for the cardiac electrophysiology-morphous merging visualization. Results and effectiveness of the proposed visualization method and discussion are shown in “Results and discussions”. And finally conclusion marks are provided in “Conclusion”.

## Data and methods

### Human cardiac cross-sectional data

The clinical general image modality such as MRI and CT are commonly used to generate 3D cardiac models [38–41], which can provide the structural and functional information of cardiac tissue and is very practical in the clinical environment. Automatic segmentation of medical images and manual correction after the segmentation process are usually needed in most cases to construct the cardiac model.

**Fig. 1:**
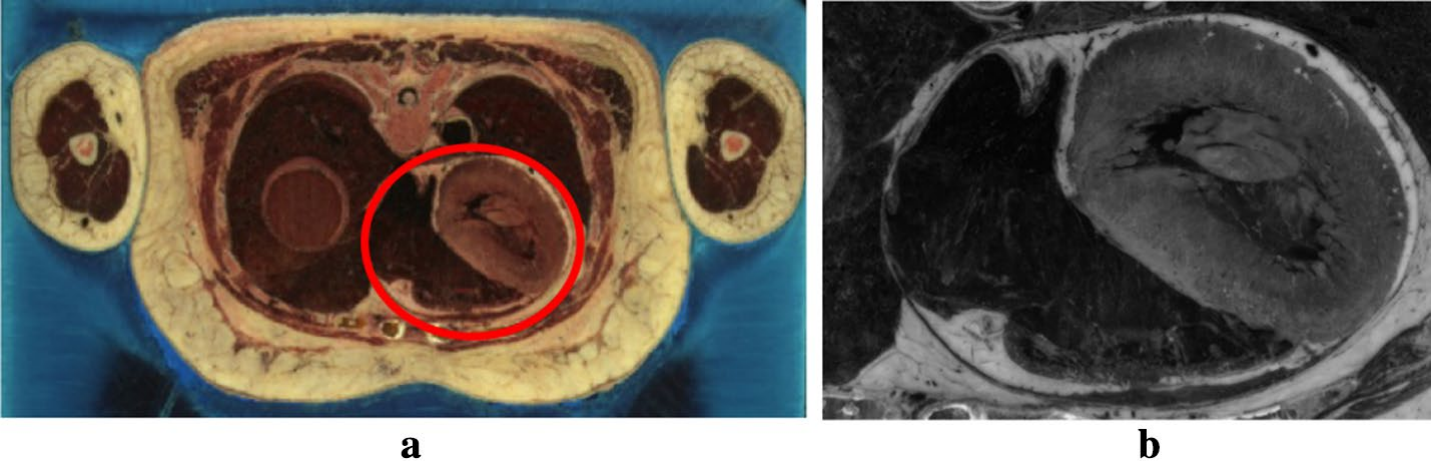
Cardiac dataset. **a** Original color cross-sectional data; **b** *Gray scale* slices

Histo-anatomical slices can also provide highly detailed anatomical and histological information [42]. In this study, the anatomical cardiac data is extracted from the cross-sectional data of National Library of Medicine’s Visible Human Project (VHP). The data including the cardiac organs are segmented out by the experts from each cross section based on anatomical features, and is converted into gray scale slices. The grayscale intensity in the slice represents various tissues within different regions in the cardiac. The raw cross-sectional data and the converted slice data are shown in Fig. 1. Figure 1a presents the female cross-sectional data which is a set of 5000 color digital images with 0.33 mm interval. There are 1280 × 2048 pixels per image and the pixel spacing in the “XY” plane is 0.33 mm. The extracted cardiac volume has 487 converted grayscale slices with the dimension of 469 × 325 in the “XY” plane. Tissues are classified by the grayscale intensity in each slice, as shown in Fig. 1b.

**Fig. 2:**
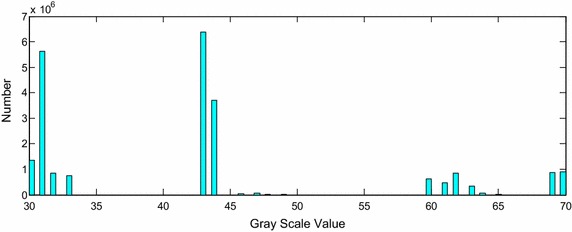
Statistical histogram of* gray* value of cardiac tissues

The statistical result of grayscale intensity of cardiac anatomy volume data is shown in Fig. 2. The horizontal axis represents the grayscale intensity corresponding to different cardiac tissues, and the vertical axis indicates the frequency of each grayscale intensity. From the statistical chart, it can be seen that the range of intensity related to the heart tissues lies in the interval between 30 and 70. Those values in this interval which are not marked on the horizontal axis are the grayscale intensities that do not appear in the data.

### Cardiac electrophysiological model

Based on the first mathematical model for the cell, the myocardial cell model can be established as follows [43]:

1 $$\frac{{dV_{m} }}{dt} = \frac{{ - (I_{ion} + I_{stim} )}}{{C_{m} }}$$

where *V*_*m*_ is the transmembrane potential and *t* is the time. *C*_*m*_ is the transmembrane capacitance per unit membrane area. *I*_*stim*_ represents stimulus current which is externally applied on the cells, and *I*_*ion*_ is the sum of all ionic currents. In this paper, according to the TNNP model [14], current *I*_*ion*_ is acquired by:

2 $$I_{ion} = I_{\text{Na}} + I_{\text{K1}} + I_{\text{To}} + I_{\text{Kr}} + I_{\text{Ks}} + I_{\text{CaL}} + I_{\text{NaCa}} + I_{\text{NaK}} + I_{\text{pCa}} + I_{\text{pK}} + I_{\text{bCa}} + I_{\text{b Na}}$$

where *I*_Na_ is fast Na^+^ current, *I*_K1_ is inward rectifier K^+^ current, *I*_to_ is transient outward current, *I*_Kr_ is rapid delayed rectifier current, *I*_Ks_ is slow delayed rectifier current, *I*_CaL_ is *L*-type Ca^2+^ current, *I*_NaCa_ is Na^+^/Ca^2+^ exchanger current, *I*_NaK_ is Na^+^/K^+^ pump current, *I*_pCa_ is plateau Ca^2+^ currents, *I*_pK_ is plateau K^+^ currents, *I*_bCa_ is background Ca^2+^ current, *I*_bNa_ is background Ca^2+^ current.

It has been reported that *I*_Ks_ density is about 2 times and *I*_to_ density is 2.0–4.5 times larger in human right ventricle than in human left ventricle. As a result, in this study, the epicardial *I*_Ks_ and *I*_to_ densities of the right ventricle are set by a factor of 2 and 4, respectively [27].

**Fig. 3:**
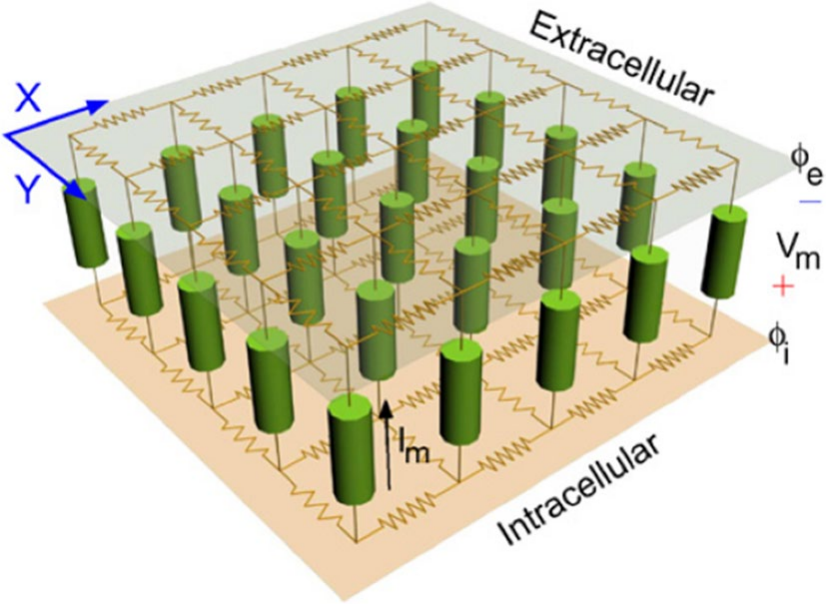
Equivalent circuit diagram of cardiac electrical conduction network [45]

Cardiac myocytes are electrically coupled to each other, and myocardial tissue is formed by the connection of cardiac myocytes through the gap junction with lower resistances. Each myocyte can thus be imagined as an individual excitable unit, which is connected with a resistor (gap junction) to form the whole cardiac conduction network [44]. Cardiac axial current flows through the resistor from one cell to another. The equivalent circuit is shown in Fig. 3. Therefore, constructing the model of cardiac tissue requires three factors: intracellular medium, extracellular medium and cell membrane.

According to the cardiac electrical conduction network, the final multicellular tissue excitation conduction model is established as following:

3 $$\frac{{dV_{m} }}{dt} = \nabla (D_{global} \nabla V{}_{m}) + \frac{{ - (I_{ion} + I_{stim} )}}{{C_{m} }}$$

$D_{global} = \frac{K}{{R_{m} C_{m} }}$, which is denoted as the diffusion tensor for describing the effective diffusion of voltage across a medium. Here $K$ is the intracellular electrical conductivity, $R_{m}$ represents the ratio of cell membrane surface area and volume.

The model in Eq. (3) is thus composed of two portions: the single cell model for describing the cellular electrical activity of cardiac cells and the intercellular electronic conductivity model for representing intercellular electronic interactions between cells. Thus the model consists of a number of ordinary differential equations (ODE) and the partial differential equation (PDE).

For the ODE, it is able to be solved by the Rush-Larsen and forward Euler method. Given a gate control variable *n*, the ordinary differential equation has the following form:

4 $$\frac{dn}{dt} = \alpha_{n} (V{}_{m}) \cdot (1-n) - \beta_{n} (V_{m} ) \cdot n$$

According to the Rush-Larsen method, let $n_{\infty } = \alpha_{n} (V_{m} )/(\alpha_{n} (V_{m} ) + \beta_{n} (V_{m} ))$ and $\tau_{n} = 1/(\alpha_{n} (V_{m} ) + \beta_{n} (V_{m} ))$, then the solution of *n* is obtained by integrating Eq. (5):

5 $$n(t) = n_{\infty } - (n_{\infty } - n_{0} ) \cdot e^{{ - t/\tau_{n} }}$$

Using the forward Euler method, numerical solution of Eq. (5) is as following:

6 $$n(t + \Delta t) = n_{\infty } - (n_{\infty } - n(t)) \cdot e^{{ - \Delta t/\tau_{n} }}$$

For the PDE in the model, its three dimensional model is as follows:

7 $$\left\{ \begin{aligned} \frac{{dV_{m} (\varsigma ,t)}}{dt} = - \frac{{I_{stim} (\varsigma ,t) + I_{ion} (\varsigma ,t)}}{{C_{m} }} + D_{global} \frac{{\partial^{2} V_{m} (\varsigma ,t)}}{{\partial \varsigma^{2} }} \hfill \\ \frac{dV(\varsigma ,0)}{dt} = V_{r} (\varsigma ) \hfill \\ \end{aligned} \right.$$

where $\varsigma$ represents the space position function *f* (*x*,*y*,*z*), $V_{r} (\varsigma )$ indicates the resting potential. To describe the electrical conduction in the human heart, the finite difference method is used to solve the cardiac dynamics of transmembrane potential equation. In this work, the forward Euler finite difference method is extended to 3D space, as in Eq. (8):

8 $$\left\{ \begin{aligned} V_{m} (x,y,z,t + \Delta t) = V_{m} (x,y,z,t){ + }\Delta t \cdot \left( - \frac{{I_{stim} + I_{ion} }}{{C_{m} }} + \hfill \right.\\ D_{global} \left(\frac{{V_{m} (x - 1,y,z,t) - 2V_{m} (x,y,z,t) + V_{m} (x + 1,y,z,t)}}{{\Delta x^{ 2} }} \right. \\ \, + \frac{{V_{m} (x,y - 1,z,t) - 2V_{m} (x,y,z,t) + V_{m} (x,y + 1,z,t)}} {{\Delta y^{ 2} }} \hfill \\ \, \left. \left. + \frac{{V_{m} (x,y,z - 1,t) - 2V_{m} (x,y,z,t) + V_{m} (x,y,z + 1,t)}} {{\Delta z^{ 2} }}\right)\right) \\ \end{aligned} \right.$$

The stability condition of the forward Euler difference equation is as follows:

9 $$\Delta t \le \frac{{\Delta \varsigma^{2} }}{{2d \cdot D_{global} }}$$

where $\Delta \varsigma$ is the spatial minimum resolution of 0.33 mm, *D*_*global*_ demonstrates the maximum electrical conductivity of 0.154 mm^2^/ms and *d* is the spatial dimension. Here, *d* = 3 for 3D ventricle models. Thus we have iterative time step Δ*t* ≤ 0.118 ms, i.e. the maximum stable time step *Δt*_*max*_ is 0.118 ms. In this paper, we use $\Delta t$ = 0.02 ms.

For the complex geometries of human ventricular tissue, precise treatment of boundary conditions would be very complicated. Many researchers have used Neumann no-flux boundary conditions for this problem [46], which is shown as Eq. (10):

10 $$n \cdot D_{global} \nabla V_{m} = 0$$

where *n* is the vector normal to the surface, means that there is zero current flow normal to the ventricular tissue boundaries.

In this paper, to solve the no-flux boundary conditions, a phase-field method is employed [47]. The major advantage of the method is that it can automatically handle the boundary conditions by introducing an auxiliary field to smooth the interface between the interior and the exterior of ventricular tissue, which decreases the complexity of computation on the premise of maintaining the accuracy. In this approach, a new variable is defined as a phase variable $\phi$, which plays a role in separating inner and outer boundary. $\phi = 0$ for those inner points and $\phi = 1$ for the outer. $\phi$ is determined by the following equation:

11 $$\frac{\partial \phi }{\partial t} = \xi^{2} \nabla^{2} \phi - \frac{\partial G(\phi )}{\partial \phi }$$

where $\xi$ is a constant for dominating the border width. When the value of $\phi$ is small enough, the no-flux boundary conditions are satisfied automatically. $G(\phi )$ is the function satisfying the condition that this function has equal minimum value when $\phi$ is either 0 or 1. In this work, we choose $G(\phi )$ as:

12 $$G(\phi ) = - \frac{{(2\phi - 1)^{2} }}{2} + \frac{{(2\phi - 1)^{4} }}{4}$$

Based on above solution process, as a result, the Eq. (1) can be substituted by:

13 $$\phi \frac{{\partial V_{m} }}{\partial t} = - \phi \frac{{I_{stim} + I_{ion} }}{{C_{m} }} + \nabla \left(D_{global} \phi \nabla V{}_{m}\right)$$

Equation (13) is integrated using the forward Euler method with ζ = 0.33 mm.

The 3D anatomical model of ventricle is constructed with 325 × 325 × 425 pixels, which is like a box includes the interior pixels i.e. the ventricular tissue points and the exterior pixels which are not ventricular tissue points. We see each interior pixel as a syncytium corresponds to ς in Eq. (7). And its space position is (x, y, z). By using the forward Euler difference method to solve Eq. (13) with ς = 0.33 mm, each syncytium’s action potential can be obtained. Combined with all syncytium’s action potential, then the electrical propagation of human ventricular tissue is formed.

### Merging attenuation function (MAF) for biophysical merging visualization

Traditional cardiac visualization methods render the heart model data through the light transport model (LTM):

14 $$L(x) = L(x_{0} )e^{{ - \int_{{x_{0} }}^{x} {\tau (t)} dt}}$$

where *x* is the position of a point t along the direction of the light, and *L*(*x*) is the light intensity at *x*. *L*(*x*_0_) represents the intensity at *x*_0_, where the ray enters the volume data. *τ*(*x*) is the absorption coefficient and depends on the location on the ray. Let $\sigma (x_{1} ,x_{2} ) = - \int_{{x_{1} }}^{{x_{2} }} {\tau (t)} dt$, which is called the optical depth, the opacity is thus defined as:

15 $$\alpha = 1 - \sigma (x_{1} ,x_{2} ) = 1 - e^{{ - \int_{{x_{{_{1} }} }}^{{x_{2} }} {\tau (t)} dt}}$$

Then the so called transfer function is introduced to map s to *L*(*s*) and to *α*(*s*), where *s* is the scalar value of a point *t* which is inside the data volume and on the ray passing through the volume. Considering all points *t* on a ray, the total amount energy along the ray can be computed by:

16 $$L = \int_{0}^{\infty } {L(x)e^{ - \sigma (0,x)} dx}$$

For the cardiac excitation conduction volume and anatomy structure volume, two linear lookup tables (LLUP) are built to implement linear mapping. Thus the respective *L*(*x*) and *α*(*s*) of voxel sample pair, i.e. *s* and it corresponding sample *s’* in the two volumes can be obtained. The linear mapping which is achieved by each LLUP is as follows:

$$\varTheta_{e} (V_{e} (x)):(\alpha_{e} (x),L_{e} (x))$$

17 $$\varTheta_{a} (V_{a} (x')):(\alpha_{a} (x'),L_{a} (x'))$$

where $V_{e} (x)$ is the action potential value and $V_{a} (x')$ is the tissue gray value. $\alpha_{e} (x)$ and $L_{e} (x)$ are opacity and light intensity of *s* in the volume of cardiac excitation conduction model respectively. $\alpha_{a} (x')$ and $L_{a} (x')$ are the analogous variables in anatomy structure volume. Thus the merging attenuation function (MAF) for the cardiac electrophysiology-morphous merging visualization is defined by:

$$\tau '(x) = \alpha_{a} (x') \cdot \alpha_{e} (x) \cdot mef$$

18 $$mef = \left\{ {\begin{array}{*{20}c} {||N(x)||} & {V_{e} (x) = - 1} \\ 1 & {other} \\ \end{array} } \right.$$

Where *mef* is the merging factor, *N*(*x*) is the normalized normal vector and ||*N*(*x*)|| is the norm of *N*(*x*). *V*_*e*_(*x*) = −1 means that only cardiac tissue data exists at current *x* position on the ray. Otherwise both cardiac conduction data and tissue data are valid at the current position.

The resultant light intensity for cardiac biophysical merging visualization is computed with the novel MAF:

19 $$L = \int_{0}^{t} {I_{merging} (x)e^{\sigma '(0,x)} dx}$$

20 $$L_{merging} (x) = \frac{{\alpha_{e} (x)}}{{\alpha_{e} (x) + \alpha_{a} (x)}}(L_{e} (x) \cdot \alpha_{e} (x)) + \frac{{\alpha_{a} (x')}}{{\alpha_{e} (x') + \alpha_{a} (x')}}(L_{a} (x') \cdot \alpha_{a} (x'))$$

Here $\alpha_{e} (x)$ and $\alpha_{a} (x')$ are the weight factors. $\sigma '$ is the merging optical depth which has the form of $\sigma '(x_{1} ,x_{2} ) = - \int_{{x_{1} }}^{{x_{2} }} {\tau '(t)} dt$.

## Results and discussions

In this section, cardiac excitation conduction model is constructed and implemented on the female cross-sectional cardiac data. Then the performance of the presented MAF based cardiac biophysical merging visualization is assessed on the same data. Experiments were carried out on a 3.70 GHz Intel E5-1620 computer with 16.0G RAM and NVIDIA Quadro k4000 graphics.

**Fig. 4:**
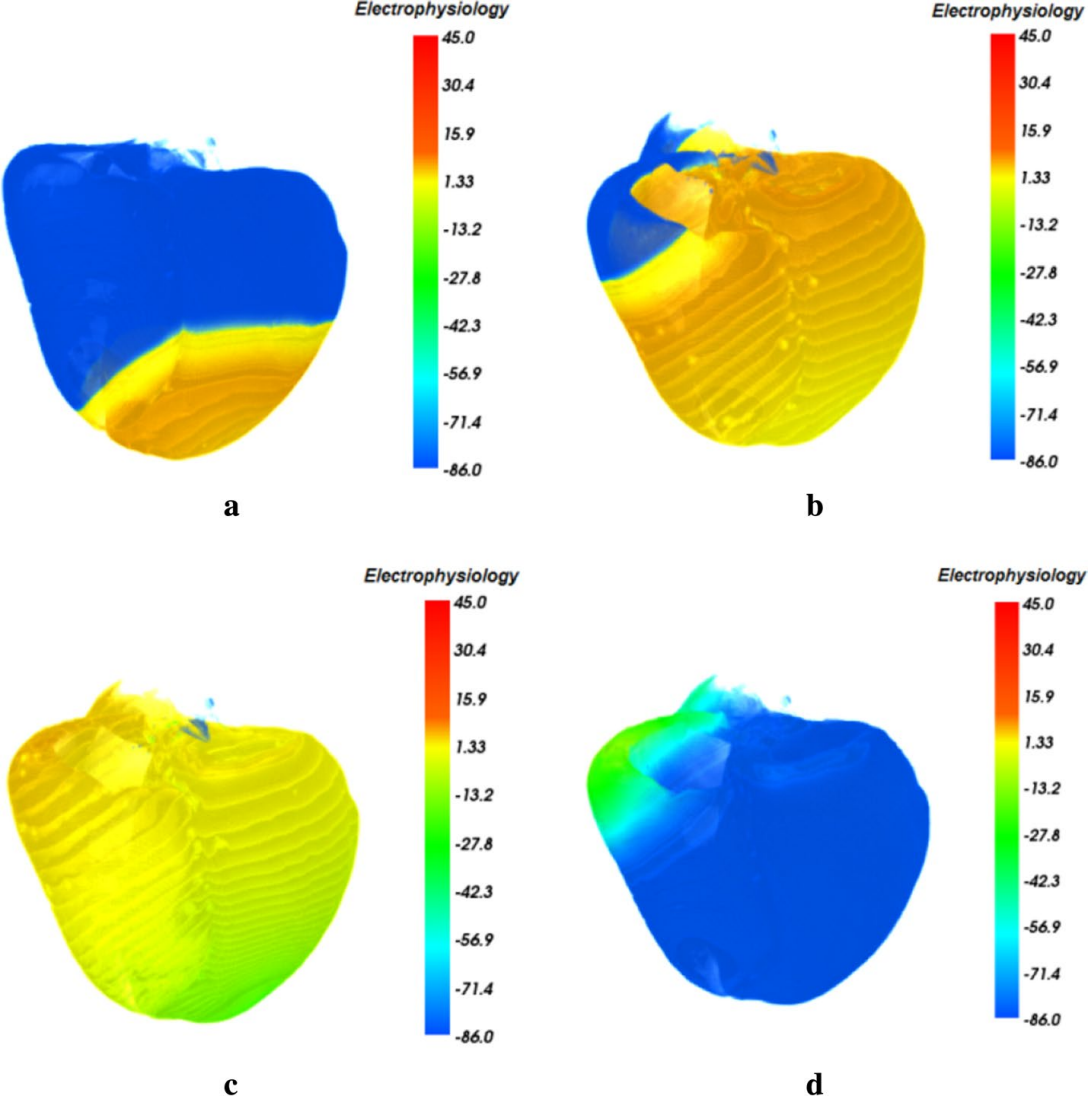
The excitation propagation of the ventricle during the excitation cycle by implementing the electrophysiological model. **a** 100 ms; **b** 200 ms; **c** 300 ms; **d** 500 ms

To explore the cardiac electrophysiological property, the cardiac excitation conduction model is constructed to describe the cardiac action potential propagation. Figure 4 shows the excitation propagation of the ventricle at different time during the excitation cycle by implementing the electrophysiological model. The propagation is visualized with the traditional light transport model in [48]. The color bar in the right of figure demonstrates the correspondence relation between the action potential of cardiomyocytes and the color. The value of −86.0 mv is the action potential of cardiomyocytes at rest state and is mapped to blue. The value of 45.0 mv which is colored by red is the peak value of action potential after depolarization. The color ranging from red to blue indicates the phases of the action potential of cardiomyocytes: depolarization and repolarization. In Fig. 4, the normal sinus excitation conduction along the ventricle is revealed. In Fig. 4a, the color of apex region in the ventricle is saffron yellow and the color of the rest region is blue at 100 ms, which means both excitations in the left ventricle and the right ventricle have propagated forward from the apex region in the phase of depolarization. In Fig. 4b, the left ventricle and most regions of the right ventricle turn to be saffron yellow at the time of 200 ms, while the base of the right ventricle still remains blue. This visualization result shows that the base of the right ventricle is the last region where the excitation propagates through during the depolarization phase. In the image of Fig. 4c, ventricle base has turned saffron yellow and the apex region changes back to green at 300 ms. The color of other regions is from green to saffron yellow. This result indicates that the action potential of cells in the ventricle starts decreasing at this time and these cells are in the repolarization phase. Figure 4d shows that most regions of the ventricle are blue except the green base part, which means much of the cells return to be the rest state in the late phase of repolarization of 500 ms.

**Fig. 5:**
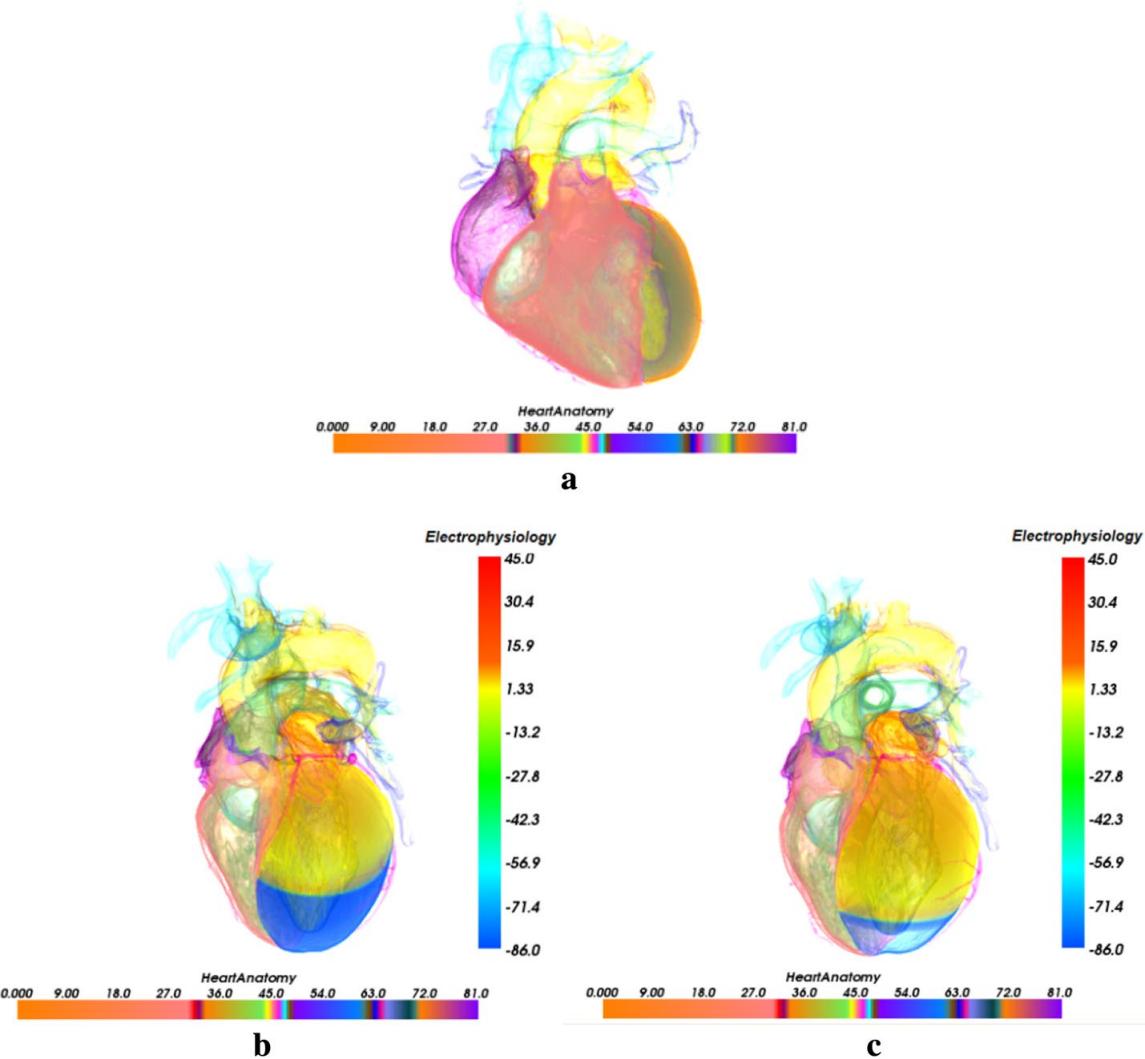
Cardiac anatomy structure visualization with traditional light transport model [44] and biophysical merging visualization of electrophysiological excitation conduction in left ventricle at different time with the context of genuine organs of heart (The* color* mapped by anatomy* bar scale* and the potential* bar scale* are not connected but separate). **a** Cardiac anatomy structure; **b** 320 ms; **c** 340 ms

Figure 5 shows the biophysically detailed visualization of the normal excitation conduction within the excitation cycle by the Merging optical model in this work. In Fig. 5a, the cardiac anatomy structure with traditional light transport model is presented. The bottom color bar gives the cardiac organs different color, e.g., the pale red organ is the right ventricle, the purple organ is the right atria, the left atria pale is colored by tan. The left ventricle close to the right ventricle and left atria is colored by cyan, and the aorta is colored by yellow. All vital organs are thus identified and distinguished without ambiguity. In Fig. 5b the action potential propagation of the left ventricle under the normal condition is coupled with the authentic fine cardiac organ anatomy, and the biophysical detailed merging cardiac is visualized. The color bar in the right of Fig. 5b is also used to map the action potential in the left ventricle to color, which is the same as in Fig. 4. The visualization result of biophysical detailed cardiac shows that the amplitude of action potential of the cells from the base to the apex area reduces gradually and the corresponding mapped color is from red to blue. This indicates that the excitation has been propagated through the left ventricle and the left ventricle is in the phase of repolarization. Thus the action potential of left ventricular cells decline after the amplitude reaches the peak value until the resting potential. Since the excitation of the cells close to the apex propagates faster than other parts [46],cells around the apex of left ventricle start repolarization firstly and their action potentials become the value of −86.0 mv. In Fig. 5c, the whole apex area has completed repolarization and is in the rest phase. From Fig. 5c, we can observe that the action potential of the cells of the apex decreases rapidly and returns to the resting potential. The corresponding color of whole apex region is blue now. While the base and the middle region of the left ventricle are in the phase of the end of the rapid repolarization at this time, and the corresponding color of these regions is from saffron yellow to cyan in the visualization result.

**Fig. 6:**
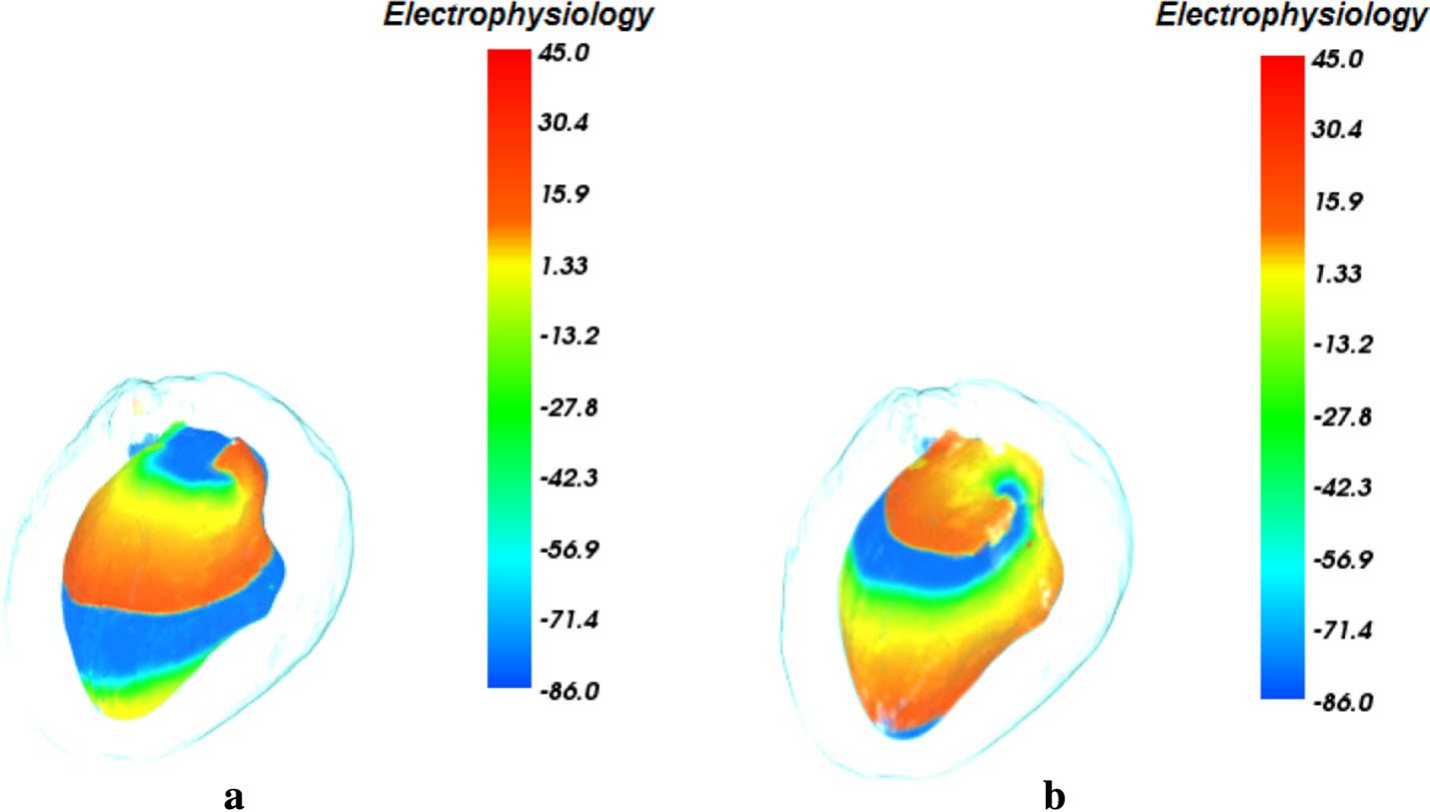
Visualization of electrophysiological re-entry in left ventricle at different time. **a** 720 ms; **b** 900 ms

Figure 6 depicts electrophysiology visualization result of re-entry propagation of the interior of the left ventricle at different time during the excitation cycle. As in Fig. 6, the highest action potential value of the cells is 45.0 mv which is colored by red. And the value of −86.0 mv also represents the value when the cells are at rest state and is mapped to blue. The value between 45.0 and −86.0 mv indicates the amplitude of action potential of the cells in the phase from the depolarization to the rest. In our experiment, to investigate and understand the re-entry propagation of the interior of the left ventricle, the opacity $\alpha_{a} (s)$ for the anatomy structure volume in the merging attenuation function is specified, which results in a more transparent left ventricle. In Fig. 6, re-entry of electrical wave in the left ventricle endocardium at the time of 720 and 900 ms is thus revealed. Since another stimulation is delivered in the left ventricle endocardium according to S1–S2 protocol [49], the balance at the tip of wavefront of the normal excitation is broken and therefore the electrical conduction speed at this position is lower than the normal conduction speed. Then the direction of excitation propagation is deflected and the re-entry, i.e. the re-entry is produced, as shown in Fig. 6a. The visualization result of Fig. 6b shows that the re-entry propagates forward stably under the normal physiological condition without any obstacle.

**Fig. 7:**
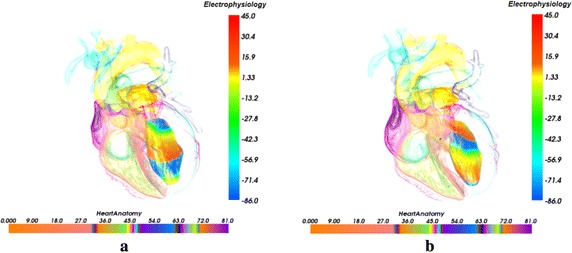
Biophysical merging visualization of electrophysiological re-entry propagation in left ventricle at different time with the context of genuine organs of heart (The* color* mapped by anatomy* bar scale* and the potential* bar scale* are not connected but separate). **a** 720 ms; **b** 900 ms

Figure 7 shows the biophysical merging visualization results of re-entry propagation of the action potential in the left ventricle with the context of genuine organs of heart. In Fig. 7, the organs of the heart and the anatomy color bar are as the same as in Fig. 5. The special position, shape, size and geometry structure of each organ are thus revealed clearly. By the proposed novel MAF, the biophysical merging of cardiac electrophysiology model of excitation conduction and anatomical model are integrated and visualized, which provides the 3D spatial position and relation of cardiac electrophysiological function process in the anatomical structure. As shown in Fig. 7a and b, a biophysically detailed heart is visualized under the normal physiological condition at different time. From the visualization results in Fig. 7a, we can see that the excitation conduction in the left ventricle endocardium starts from the apex of left ventricle and the conducting space is in the left front of the heart. The re-entrant in left ventricle endocardium propagates forward smoothly from the apex to the base area at the time of 720 ms during repolarization, which indicates that the cardiac electrophysiological state becomes stable after depolarization. At 900 ms, the apex area has been fully repolarized and arrived to the resting phase. Since the potential distribution and electrophysiological activity in the 3D space within the heart can be observed intuitively, it is more convenient and reliable for researchers to analyze the functional effects of pathological factors such as myocardial ischemia on the propagation of the reentry wave.

The time consumption of the proposed method includes the time of computing cardiac excitation conduction model data and implementing the merging visualization of this data and cardiac structure data with the proposed merging attenuation function. Computing cardiac ventricle excitation conduction model data took 2 min 40 s for the simulation at a certain moment within the excitation cycle. The merging visualization for the ventricle structure data which has the size of 325 × 325 × 425 took 1 min 30 s. The total consuming time is 4 min 10 s. With the increased degree of the complication of the image set, the proposed method will take more computing time. When we implemented the merging visualization method on the whole heart geometry data of 469 × 325 × 487, it took 2 min 50 s to produce the heart image. The total consuming time became 5 min 30 s.

Since it is very difficult to observe the cardiac electrical activities in the tissue level, generally the open chest surgery is carried out and an electrode array is placed on the heart surface to monitor the electrical activities. However the experiment in vivo is infeasible for mankind. As an alternative method, a large number of animal patch clamping experiments were used. Based on the experimental data, the computational models were built to simulate the above activities. Here some classical and authoritative articles are available that cover models from cellular electrophysiology to models of cardiac anatomy. In our manuscript, the TNNP06 model is used, which can in detail describe different aspects of human ventricular electrophysiological function and dysfunction. It is constructed with 12 major ionic currents, and fits the experimentally measured APDR properties of human myocardium and the CVR properties for currently only available for dog and guinea pig model and so on. The model is also able to reproduce the APs of endo-, m-, and epimyocardial regions of the ventricles and their different rate dependencies. The methodology in this work is implemented on the electrophysiological data generated by the TNNP06 model and the real anatomical data. Therefore it can explore the cardiac action potential and other necessary electrophysiological characteristics which fit the experimental data.

## Conclusion

In this paper, we introduce a novel exploratory method for cardiac biophysical merging visualization of biophysical coupling of electrophysiological activity and cardiac anatomy structure. Compared to previous methods, the proposed approach comprehensively highlights certain features of interest of cardiac electrophysiological property in the authentic fine organ anatomy context. In order to explore detailed cardiac electrical activity, firstly a cardiac electrical excitation propagation model is implemented on the cardiac cross-sectional data and the volume of cardiomyocytes electrical excitation conduction is obtained. Then the MAF is constructed to integrate cardiac anatomy with electrophysiological volume and visualize a biophysically detailed merging heart.

The method presented in this work is a useful way for providing detailed electrophysiological behavior information while delivering detailed anatomical circumstance, which allows researchers to explore intricate functional mechanisms, which can further help speculate cardiac physiological and pathological properties for cardiac research and clinical diagnoses. Practical applications of our work will be the platform for having insights into cardiac disorders such as mechanisms of arrhythmia, and accordingly be the tools to aid in the prevention, diagnosis and treatment of cardiac diseases.

## Abbreviations

LTM: light transport model
MAF: merging attenuation function
LLUT: linear look up table

## References

[1] Gray RA, Pertsov AM, Jalife J (1998). Spatial and temporal organization during cardiac fibrillation. Nature.

[2] Noble D (2002). Modeling the heart-from genes to cells to the whole organ. Science.

[3] Hunter P, Smith N, Fernandez J, Tawhai M (2005). Integration from proteins to organs: the IUPS Physiome Project. Mech Ageing Dev.

[4] Keller DUJ, Weber FM, Seemann G, Dössel O (2010). Ranking the influence of tissue conductivities on forward-calculated ECGs. IEEE Trans Biomed Eng.

[5] Serpooshan V, Zhao M, Wei SAK, Shah PB, Wang A, Mahmoudi M, Malkovskiy AV, Rajadas J, Butte MJ (2014). Use of bio-mimetic three-dimensional technology in therapeutics for heart disease. Bioengineered.

[6] Zhang L, Wang K, Zheng F, Li X, Luo S (2014). 3D Cardiac microvessels embolization imaging based on X-ray phase contrast imaging. Biomed Eng Online..

[7] Taimouri V, Hua J (2013). Visualization of shape motions in shape space. IEEE Trans Visual Comput Graph.

[8] Spicher N, Kukuk M, Maderwald S, Ladd ME (2016). Initial evaluation of prospective cardiac triggering using photoplethysmography signals recorded with a video camera compared to pulse oximetry and electrocardiography at 7T MRI. Biomed Eng Online..

[9] Noble D (1962). A modification of the HodgkineHuxley equations applicable to Purkinje fibre action and pace-maker potentials. J Physiol.

[10] Zhang H, Holden AV, Kodama I (2000). Mathematical models of action potentials in the periphery and center of the rabbit sinoatrial node. Am J Physiol.

[11] Beeler GW, Reuter H (1977). Reconstruction of the action potential of ventricular myocardial fibres. J Physiol.

[12] Luo C, Rudy Y (1994). A dynamic-model of the cardiac ventricular action-potential: I. Simulations of ionic currents and concentration changes. Circ Res.

[13] Priebe L, Beuckelmann DJ (1998). Simulation study of cellular electrical properties in heart failure. Circ Res.

[14] ten Tusscher KHWJ, Panfilov AV (2006). Alternans and spiral breakup in a human ventricular tissue model. Am J Physiol Heart Circ Physiol..

[15] Hilemann DW, Noble D (1987). Excitation-contraction coupling and extracellular calcium transients in rabbit atrium: reconstruction of basic cellular mechanisms. Proc R Soc Lond B.

[16] Nygren A, Fiset C, Firek L (1998). Mathematical model of an adult human atrial cell: the role of K+ currents in repolarization. Circ Res.

[17] Courtemanche M, Ramirez RJ, Nattel S (1998). Ionic mechanisms underlying human atrial action potential properties: insights from a mathematical model. Am J Physiol.

[18] Nygren A, Leon LJ, Giles WR (2001). Simulations of the human atrial action potential. Philos Trans R Soc Lond A..

[19] Kneller JR, Sun H, Leblanc N, Nattel S (2002). Remodeling of Ca^2+^-handling by atrial tachycardia: evidence for a role in loss of rate-adaptation. Cardiovasc Res.

[20] Michailova A, DelPrincipe F, Egger M, Niggli E (2002). Spatiotemporal features of Ca^2+^ buffering and diffusion in atrial cardiac myocytes with inhibited sarcoplasmic reticulum. Biophys J.

[21] Salinet JL, Masca N, Stafford PJ, Ng GA, Schlindwein FS (2016). Three-dimensional dominant frequency mapping using autoregressive spectral analysis of atrial electrograms of patients in persistent atrial fibrillation. Biomed Eng Online..

[22] Yang F, Lu WG, Zhang L, Zuo WM, Wang KQ, Zhang HG. Fusion visualization for cardiac anatomical and ischemic models with depth weighted optic radiation function. Computing in Cardiology Conference. 2015. pp 937–940.

[23] Yang F, Zhang L, Lu W (2016). Depth attenuation degree based visualization for cardiac ischemic electrophysiological feature exploration. Biomed Res Int.

[24] Zhang L, Gai C, Wang K, Lu W, Zuo W. GPU-based high performance wave propagation simulation of ischemia in anatomically detailed ventricle. In: Computer in cardiology. 2011. pp 469–472.

[25] Rubio-Guivernau JL, Perez-David E, Arenal Á, Bermejo J, Santos A, Ledesma-Carbayo MJ (2011). 3D visualization of myocardial substrate using Delayed Enhancement MRI for pre-planning and guidance of ablation procedures of ventricular tachycardia. J Cardiovasc Magn Reson.

[26] Lu W, Li J, Yang F, Wang K (2015). Simulation study of ventricular arrhythmia at the early stage of global ischemic condition. Progr Biochem Biophys..

[27] Lu W, Li J, Yang F, Luo C, Wang K, Adeniran I, Zhang H (2015). Effects of acute global ischemia on re-entrant arrhythmogenesis: a simulation study. J Biol Syst..

[28] Yang F, Zhang L, Lu W, Zuo W, Wang K, Zhang H, Li Y (2014). Multivariate cardiac data visualization based on multi-dimensional transfer function with ray distance. Bio-Med Mater Eng.

[29] Zhang L, Gai C, Wang K, Zuo W. Real-time interactive heart illustration platform via hardware accelerated rendering. In: International conference on advanced computer control. 2011. pp 497–501.

[30] Wang K, Zhang L, Gai C, Zuo W. Illustrative visualization of segmented human cardiac anatomy based on context-preserving model. In: Computer in cardiology. 2011. pp 485–488.

[31] Gai C, Wang K, Zhang L, Zuo W (2012). Strategy of statistics-based visualization for segmented 3d cardiac volume data set. Adv Intell Comput..

[32] Zhang L, Wang K, Zhang H, Zuo W, Liang X, Shi J (2014). Illustrative cardiac visualization via perception-based lighting enhancement. J Med Imag Health Inform..

[33] Zhang L, Wang K, Yang F, Lu W, Wang K, Zhang Y, Liang X, Han D, Zhu Y (2016). A visualization system for interactive exploration of the cardiac anatomy. J Med Syst.

[34] Seemann G (1843). Heterogeneous three-dimensional anatomical and electro-physiological model of human atria. Philos Transact A Math Phys Eng Sci..

[35] Zhang L, Wang K, Zuo W, Wu Y, Han D. GPU-based fusion method for 3D electrophysiological data visualization. In: Proc. International conference on computerized healthcare. 2012. pp 51–56.

[36] Zhang L, Wang K, Zuo W, Yang M (2013). Real-time multi-volume rendering for 3D electrophysiological data visualization based on graphics processing unit. ICIC Expr Lett Appl..

[37] Zhang L, Wang K, Zuo W, Gai C (2014). G-Heart: a GPU-based system for electrophysiological simulation and multi-modality cardiac visualization. J Comput..

[38] Virag N, Jacquemet V, Henriquez CS, Zozor S, Blanc O, Vesin JM (2002). Study of atrial arrhythmias in a computer model based on magnetic resonance images of human atria. Chaos.

[39] Helm PA, Tseng HJ, Younes L, McVeigh ER, Winslow RL (2005). Ex vivo 3D diffusion tensor imaging and quantification of cardiac laminar structure. Magn Reson Med.

[40] Gurev V, Lee T, Constantino J, Arevalo H, Trayanova NA (2011). Models of cardiac electromechanics based on individual hearts imaging data: image-based electromechanical models of the heart. Biomech Model Mechanobiol.

[41] Aslanidi OV, Nikolaidou T, Zhao J, Smaill BH, Gilbert SH, Holden AV (2013). Application of micro-computed tomography with iodine staining to cardiac imaging, segmentation, and computational model development. IEEE Trans Med Imaging.

[42] Zhao J, Butters TD, Zhang H, LeGrice IJ, Sands GB, Smaill BH (2013). Image-based model of atrial anatomy and electrical activation: a computational platform for investigating atrial arrhythmia. IEEE Trans Med Imaging.

[43] Nobel D (1960). Cardiac action and pacemaker potentials based on the Hodgkin–Huxley equations. Nature.

[44] Latimer D, Roth B (1998). Electrical stimulation of cardiac tissue by a bipolar electrode in a conductive bath. IEEE Trans Biomed Eng.

[45] Vigmond EJ, Weber SR, Prassl AJ, Deo M, Plank G (2008). Solvers for the cardiac bidomain equations. Prog Biophys Mol Biol.

[46] Clayton RH, Panfilov AV (2008). A guide to modelling cardiac electrical activity in anatomically detailed ventricles. Prog Biophys Mol Biol.

[47] Fenton FH, Cherry EM, Karma A, Rappel WJ (2005). Modeling wave propagation in realistic heart geometries using the phase-field method. Chaos..

[48] Max N (1995). Optical models for direct volume rendering. IEEE Trans Visual Comput Graph.

[49] Keller DUJ, Kalayciyan R, Dössel O, Seemann G (2010). Fast creation of endocardial stimulation profiles for the realistic simulation of body surface ECGs. IFMBE Proc..

